# Predicting fluid responsiveness in ICU patients: comparison of different parameters and cutoff limits using pulse power analysis assessment

**DOI:** 10.1186/cc14260

**Published:** 2015-03-16

**Authors:** H Barrasa, J Maynar, S Castaño, Y Poveda, P Garcia Domelo, A Tejero, G Baziskueta, A Quintano, B Fernández Miret, M Iturbe, S Cabañes, F Fonseca

**Affiliations:** Alava University Hospital-Santiago, Vitoria, Spain

## Introduction

Dynamic parameters are becoming standard for fluid responsiveness assessment. Cutoff values are different in the literature. The aim was to assess the accuracy of different preload parameters to predict fluid responsiveness using pulse power analysis and to compare different levels of hemodynamic response due to passive leg raising (PLR) against the effect of a fluid challenge (FC).

## Methods

A prospective study in a 17-bed mixed ICU. Patients were fully ventilated and CO monitored with LiDCOplus® and underwent a FC due to hypotension and/or hypoperfusion and preload dependence (SVV and/or PPV >10%). PLR was performed before FC. Hemodynamic data were recorded prePLR, postPLR and postFC with 0.5 l crystalloids. We compared different cutoff values of increase in CO and SV (10 to 15%) to assess the ability of PLR, SVV, PPV and CVP to predict the response to FC. Statistical analysis: continuous variables expressed as mean ± SD. Comparison before and after was done using a paired Student's *t* test, and receiver operating characteristic (ROC) curves were generated by varying the discriminating threshold of each variable.

## Results

Thirty-one patients were included. Baseline parameters: MAP 70.5 mmHg (SD 13.3) 87% under catecholamine, SV 55.32 ml (SD 20.2), CO 5.2 l (SD 2), SVV 16.8% (SD 12), PPV 19.1% (SD 14), HR 96 bpm (SD 18) and CVP 9.2 mmHg (SD 2.5). In total, 41.9% of patients increased 15% CO after FC (selected as responders), 38.7% after the PLR. Differences in responders versus nonresponder patients were: baseline SVV (23.9 vs. 11.6; *P* = 0.02) and PPV (28.4 vs. 12.4; *P* = 0.01). Differences in SV and CO were not statistically significant. The best parameter to predict positive response to FC was PLR with cutoff 12.6% for CO increase: sensitivity 84.6% (95% CI = 65 to 104), specificity 94.4% (95% CI = 84 to 105) and AU ROC 0.94 (95% CI = 0.86 to 1.0). ROC was also good for SVV 0.835 (95% CI = 0.66 to 1.0; *P* = 0.002) and PPV 0.833 (95% CI = 0.681 to 0.985; *P* = 0.002) in this cutoff value. In SV increase, PLR, SVV and PPV had *P* < 0.05, but with worse ROC. In addition, SVV <13% identified patients who will not increase MAP with FC: sensitivity 91.7% (95% CI = 76 to 107.3%), negative predictive value 93.5 (95% CI = 80.7 to 106). CVP failed to distinguish responders from nonresponders.

## Conclusion

Our results support the idea that a reversible FC (PLR; CO cutoff 12.6%) is best at identifying responder patients to a FC. Dynamic parameters (SVV/PPV) are also effective when appropriate. Beat-tobeat SV and CO using pulse power analysis is a valid tool for these tests.

